# Several cases of arterial thrombosis of the limbs revealing asymptomatic and paucisymptomatic COVID-19 infection

**DOI:** 10.11604/pamj.2021.38.209.28188

**Published:** 2021-02-24

**Authors:** Ibrahima Niang, Daouda Thioub, Mamadou Ly

**Affiliations:** 1 Department of Radiology, Fann University Hospital Center, Dakar, Senegal,; 2 Department of Infectious Diseases, Fann University Hospital Center, Dakar, Senegal

**Keywords:** COVID-19, arterial thrombosis, Africa, Senegal, emergency

## To the editors of the Pan African Medical Journal

The COVID-19 pandemic, which has been in progress for more than a year, has consistently demonstrated its multi-systemic nature, with its varied effects affecting all the body's systems [1]. The cardiovascular system is far from being an exception. And the main cardiovascular events reported, associated with COVID-19, concern venous thromboembolic diseases (VTED) [2, 3]. While VTED can quickly become life-threatening, arterial thrombosis of the limbs is just as urgent and deleterious because of the risk of compromising the functional prognosis, which can lead to amputation [2, 3]. Indeed, COVID-19 infection leads to blood hypercoagulability, which causes arterial and venous thrombosis at the peripheral (upper and lower limbs) and central (cerebral, pulmonary, coronary...) levels [4]. This high risk of thrombosis is now known in patients diagnosed with COVID-19, who are receiving anticoagulant treatment combined with other preventive measures to limit thromboembolic events.

However, patients who have asymptomatic or paucisymptomatic forms of undiagnosed COVID-19, and therefore do not have preventive anticoagulant therapy or clinical monitoring, are at high risk of developing arterial thrombosis [5]. When the diagnosis of arterial thrombosis of the lower limbs is most often delayed, the high risk of these patients to have an amputation becomes apparent. So, the first challenge for these patients is to diagnose arterial thrombosis. This challenge is compounded by the fact that these patients may have no prior risk factors for thrombosis. Therefore, if the vascular examination is not properly conducted and the arterial ultrasound Doppler is not promptly prescribed, the functional prognosis of the limb is quickly engaged. And once the diagnosis of arterial thrombosis is established, the second challenge remains to link it to a COVID-19 infection, as the sensitivity of the polymerase chain reaction (PCR) test is not always optimal [6]. Moreover, it is also possible that the thrombotic event may occur after the time of PCR test positivity, hence the interest of using a serological test to prove current or past SARS-CoV-2 infection (IgM+ or IgG+).

**Figure 1:**
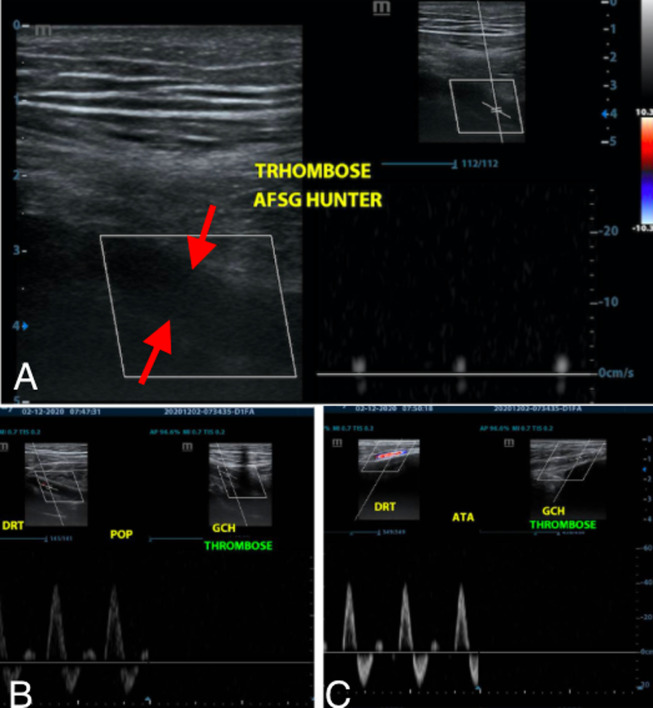
example of a Doppler ultrasound scan of a 30-year-old patient with no known pathological condition, received for pain in the left lower limb for 3 days; the ultrasound doppler allowed the diagnosis of arterial thrombosis of the left lower limb involving the femoral; (A) popliteal; (B) and tibial; (C) with a hypoechogenic occlusive thrombus (delimited by the red arrows); in this patient, despite the absence of symptoms suggestive of COVID-19, the PCR test was positive

We are thus alerting on several cases of patients without any particular history received as outpatients in the emergency department of the Fann University Hospital (Dakar-Senegal) for symptoms including pain and swelling of limbs (upper or lower) and in whom the diagnosis of arterial limb thrombosis has been established by the ultrasound doppler (Figure 1). The rapidity to make this diagnosis and to treat these patients by thrombectomy and effective anticoagulation is essential for the vital but above all functional prognosis. In conclusion, we would like to draw the attention of caregivers to the increase of these cases of arterial thrombosis with no a priori risk factor. Their diagnosis should not suffer any delay and their link with COVID-19 should be established and lead to a serological test even if the PCR is negative.
